# Effects of Exercise on Flow-Mediated Dilation in Patients with Heart Failure: A Systematic Review and Meta-Analysis of Randomized Controlled Trials

**DOI:** 10.3390/jcdd12120458

**Published:** 2025-11-25

**Authors:** Yongjie Chen, Bing Han, Yifan Zhang, Boya Gu, Yuanyuan Lv, Laikang Yu

**Affiliations:** 1 Beijing Key Laboratory of Sports Performance and Skill Assessment, Beijing Sport University, Beijing 100084, China; youngj@bsu.edu.cn; 2 Department of Strength and Conditioning Assessment and Monitoring, Beijing Sport University, Beijing 100084, China; hanbing02@bsu.edu.cn (B.H.); 18738314013@163.com (Y.Z.); 3 Beijing Anti-Doping Laboratory, Beijing Sport University, Beijing 100084, China; guboya@sohu.com; 4 China Institute of Sport and Health Science, Beijing Sport University, Beijing 100084, China

**Keywords:** heart failure, endothelial function, flow-mediated dilation, aerobic exercise

## Abstract

This study aimed to evaluate the effects of exercise on vascular flow-mediated dilation (FMD) in patients with heart failure (HF) and to identify the optimal exercise model for this population. A comprehensive search was conducted in the Web of Science, Scopus, PubMed, Embase, and Cochrane databases, including data published up to 18 August 2025. A meta-analysis was conducted to calculate standardized mean difference (SMD) and 95% confidence interval. Eleven studies met the inclusion criteria, comprising 224 participants in the intervention groups and 185 participants in the control groups. The results demonstrated that exercise significantly improved FMD (SMD = 1.14, *p* < 0.0001). Subgroup analysis showed that aerobic exercise (SMD = 1.25, *p* < 0.0001), intervention period ≤ 8 weeks (SMD = 2.19, *p* < 0.00001) Intervention frequency > 3 times per week (SMD = 2.82, *p* < 0.00001) and each intervention duration < 60 min (SMD = 1.22, *p* = 0.01) were the most effective in improving FMD in patients with HF. This meta-analysis indicates that aerobic exercise performed more than three times per week, for sessions under 60 min and over an intervention period of up to 8 weeks, is associated with meaningful improvements in FMD in HF patients. These findings offer clear and actionable guidance for clinicians when prescribing exercise to support vascular health in this population.

## 1. Introduction

Heart failure (HF) is a multifaceted clinical syndrome characterized by progressive deterioration of ventricular function. It has become an increasingly severe global health challenge, with substantial clinical and socio-economic implications. Current epidemiological estimates indicate that more than 64 million people worldwide are affected by HF, and this prevalence is projected to rise further due to population aging and improved survival following cardiovascular events [1]. Despite advances in pharmacological and interventional therapies, the 5-year mortality rate of HF remains approximately 50%, a figure comparable to that of many malignant tumors [2]. Consequently, HF warrants continued and urgent attention from both clinical and research perspectives.

The central pathophysiological feature of HF is impaired ventricular filling or ejection capacity, which results in diminished cardiac output. Insufficient cardiac output compromises the ability to meet systemic metabolic demands during exercise, thereby contributing to exercise intolerance (typically manifested as dyspnea and fatigue) and fluid retention [3,4]. Distinct subtypes of HF present with different mechanisms: HF with reduced ejection fraction (HFrEF) is characterized by impaired myocardial contractility, often accompanied by left ventricular dilation and excessive neurohormonal activation [4], whereas HF with preserved ejection fraction (HFpEF) is primarily associated with abnormal diastolic function, increased myocardial stiffness, and left ventricular hypertrophy, often driven by hypertension and metabolic syndrome [5,6].

Vascular endothelial dysfunction constitutes a key pathological mechanism in HF, contributing to increased vascular resistance, heightened inflammation and oxidative stress, and the amplification of the HF vicious cycle [7]. It also serves as a strong independent predictor of rehospitalization and mortality in HF patients [8], highlighting the importance of accurately assessing endothelial function in disease management. FMD, recognized as the noninvasive gold standard for evaluating endothelial function [9,10], has also been identified as an independent prognostic indicator in chronic HF [11]. Katz et al. [12] reported that baseline FMD is markedly reduced in HF patients, with those exhibiting severe impairment demonstrating higher rates of all-cause mortality and heart transplantation. Even under standard pharmacological therapy, most HF patients maintain FMD values below those of healthy individuals, reflecting a persistent “residual risk” associated with vascular dysfunction [11,13] and underscoring the need for complementary targeted interventions. In this context, regular exercise training has attracted growing clinical interest as a highly effective non-pharmacological strategy.

Exercise is widely recognized as an effective non-pharmacological intervention to enhance endothelial function in HF patients. The 2013 American College of Cardiology Foundation/American Heart Association (ACCF/AHA) guidelines recommend individualized exercise programs, such as walking and cycling, to improve endothelial function and prognosis in HF, while at least 150 min of moderate-intensity weekly exercise is advised for healthy individuals to maintain vascular health and reduce cardiovascular risk [14]. For example, Van Craenenbroeck et al. [15] reported that six months of exercise intervention improved circulating angiogenic cell function and increased CD34+/KDR+ endothelial progenitor cells in chronic HF patients, alongside a 28% improvement in FMD. Similarly, Fuertes-Kenneally et al. [16] demonstrated that exercise-based cardiac rehabilitation (CR) significantly improved endothelial function in HF patients, suggesting potential mortality reduction and enhanced health benefits.

Nevertheless, the evidence is not unequivocal. Some studies have failed to demonstrate significant improvements in endothelial function following exercise interventions. Benda et al. [17] observed no significant effect of 12 weeks of exercise intervention on FMD, venous vascular function, and quality of life in HF patients. Likewise, Kitzman et al. [18] reported that 16 weeks of endurance training significantly increased peak oxygen uptake (VO_2_peak) in older HF patients but did not improve brachial artery FMD or arterial stiffness. Turri-Silva et al. [19] found that high-intensity interval training was superior to resistance training in enhancing cardiopulmonary fitness, muscle strength, and functional performance, yet neither modality significantly improved endothelial function.

Previous meta-analyses examining the effects of exercise on vascular endothelial function in HF patients have also been limited by methodological heterogeneity. For instance, Pearson et al. [20] included interventions such as functional electrical stimulation (FES) and inspiratory muscle training (IMT) under the umbrella of exercise intervention, diverging from conventional definitions of structured exercise. Similarly, Early et al. [21] incorporated studies involving non-HF populations (e.g., patients with hypercholesterolemia, overweight and obese children, and healthy participants). In the meta-analysis by Fuertes-Kenneally et al. [16,22], some control groups also received exercise interventions, complicating interpretation of the findings.

Therefore, the present study aims to systematically evaluate the effect of exercise on FMD in HF patients, with particular emphasis on identifying optimal exercise modalities to improve vascular endothelial function.

## 2. Materials and Methods

This systematic review and meta-analysis were conducted in accordance with the Preferred Reporting Item for Systematic Reviews and Meta-Analyses (PRISMA) guidelines. The study protocol was prospectively registered in PROSPERO (CRD42024507099).

### 2.1. Search Strategy

A comprehensive search was performed in Web of Science, Scopus, Embase, Cochrane Library, and PubMed from database inception to 18 August 2025. Search terms included a combination of keywords and Medical Subject Headings (MeSH) related to exercise, vascular endothelial function, and heart failure (Table S1). Screening was independently conducted by the two authors (Y.C. and B.H.). In cases of disagreement, a third author (L.Y.) adjudicated until consensus was reached.

### 2.2. Eligibility Criteria

The Population, Intervention, Comparison, Outcome, Study design (PICOS) framework was used to define the inclusion. (1) Population: participants with a clinical diagnosis of HF; (2) Intervention: participants randomly assigned to either the intervention group or control group; (3) Comparison: studies that measured endothelial function before and after the intervention; (4) Outcome: the primary outcome was FMD; and (5) Study design: randomized controlled trial (RCT) design.

### 2.3. Exclusion Criteria

Exclusion criteria were: (1) non-English publications; (2) conference abstracts; (3) review articles; (4) studies with outcomes that could not be converted into mean and standard deviation (SD); and (5) studies in which the control group received exercise interventions.

### 2.4. Data Extraction

Two authors (Y.C. and B.H.) independently extracted data using a standardized extraction form. Discrepancies were resolved through discussion and, if necessary, a second round of verification. Extracted information included: study characteristics (first author, publication year), sample size, participant demographics (age, gender, HF severity), details of exercise interventions (type, intensity, session duration, frequency, overall intervention length), and mean ± SD values of primary outcomes.

### 2.5. Methodological Quality Assessment

The methodological quality of included studies was assessed using the Cochrane Risk of Bias Tool (RoB) [23]. This tool evaluates seven domains: random sequence generation (selection bias), allocation concealment (selection bias), blinding of participants and personnel (performance bias), blinding of outcome evaluation (detection bias), incomplete outcome data (depletion bias), selective reporting (reporting bias), and other biases [24]. Each study was rated as having low, uncertain or high risk of bias across these domains. Two authors (Y.C. and B.H.) conducted assessments independently, with disagreements resolved through consultation with a third author (L.Y.).

### 2.6. Certainty Assessment

The Grading of Recommendations Assessment, Development and Evaluation (GRADE) approach was used to assess the certainty of evidence across outcomes, with ratings categorized as high, moderate, low, or very low. The GRADE assessment was conducted by two independent authors (Y.C. and B.H.).

### 2.7. Statistical Analysis

For meta-analysis, mean values and SDs were extracted from each included study. When outcomes were reported as standard error of the mean (SE), values were converted to SDs. Pooled effect sizes were calculated using a random-effects model, with SMDs and 95% CIs reported [25,26]. Statistical heterogeneity was quantified using the I^2^ statistic, with thresholds defined as follows: I^2^ < 25% (no heterogeneity), 25–50% (low heterogeneity), 50–75% (moderate heterogeneity), and >75% (high heterogeneity) [27]. Forest plots were generated using RevMan 5.4 software, while meta-regression, sensitivity analysis, and funnel plot were produced using Stata 17. Statistical significance was defined as *p* < 0.05.

## 3. Results

### 3.1. Study Selection

**Figure 1:**
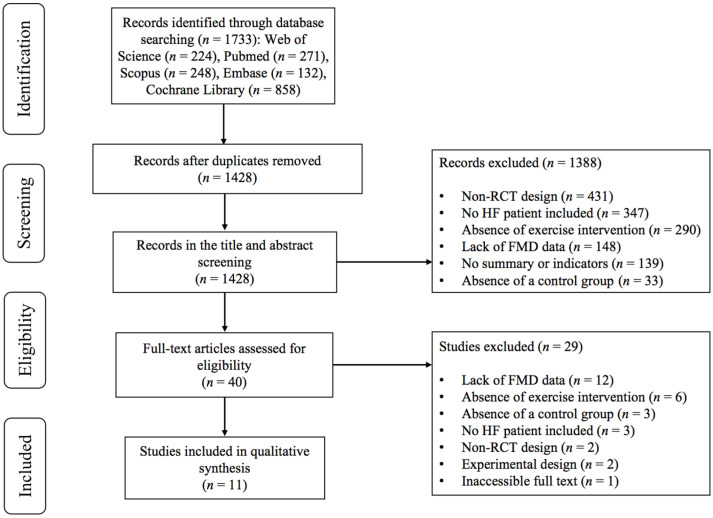
PRISMA flowchart of study selection.

As illustrated in Figure 1, the initial database search yielded 1733 records. After removing duplicates, 1428 remained. Following title and abstract screening, 1399 studies were excluded for not meeting the inclusion criteria. Full-text review led to the exclusion of an additional 29 studies for the following reasons: (1) lack of FMD data (*n* = 12); (2) absence of exercise intervention (*n* = 6); (3) absence of a control group (*n* = 3); (4) no HF patients included (*n* = 3); (5) non-RCT design (*n* = 2); (6) experimental design (*n* = 2); and (7) inaccessible full text (*n* = 1). Ultimately, 11 studies [15,18,19,28,29,30,31,32,33,34,35] met the inclusion criteria and were retained for further analysis.

### 3.2. Characteristics of Included Studies

Table S2 provides an overview of the included studies, including 224 participants in the intervention groups and 185 participants in the control groups. Two studies recruited patients with NYHA class I-II HF [19,30], while the remaining 9 studies [15,18,28,29,31,32,33,34,35] included patients with NYHA II-IV. Four studies [19,28,34,35] employed multiple intervention groups. All intervention groups received exercise intervention, whereas control groups received either placebo or standard care interventions (e.g., daily life activities, medication, routine care). Intervention durations ranged from 6 to 24 weeks, with session durations varying from 5 to 60 min. All studies assessed brachial artery FMD, primarily via high-resolution Doppler ultrasound.

### 3.3. Meta-Analysis

**Figure 2:**
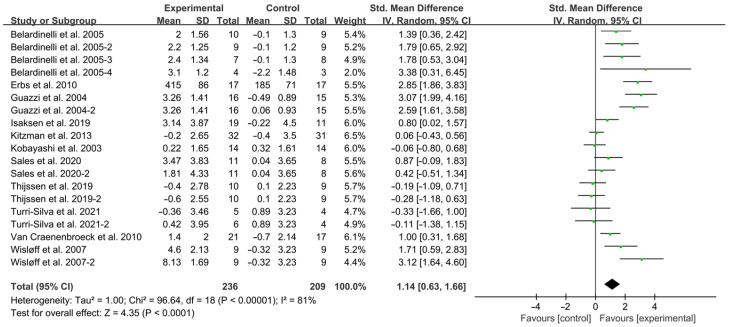
Meta-analysis results of the effects of exercise on FMD in HF patients [15,18,19,28,29,30,31,32,33,34,35].

As shown in Figure 2, exercise significantly improved FMD in HF patients (SMD = 1.14; 95% CI: 0.63 to 1.66, *p* < 0.0001, I^2^ = 81%).

### 3.4. Meta-Regression

As shown in Table S3, no significant correlations were found between intervention duration (*p* = 0.133) or session duration (*p* = 0.785) and FMD. However, frequency (*p* = 0.007) and weekly time (*p* = 0.035) were significantly associated with improvements in FMD.

### 3.5. Subgroup Analysis

**Figure 3:**
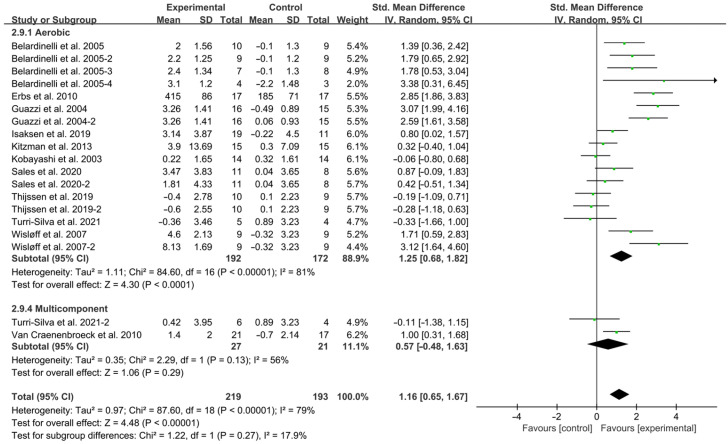
Meta-analysis results of the effects of intervention type on FMD in HF patients [15,18,19,28,29,30,31,32,33,34,35].

Aerobic exercise significantly improved FMD in HF patients (SMD = 1.25; 95% CI: 0.68 to 1.82, *p* < 0.0001, I^2^ = 81%) while multicomponent training showed no significant effect (SMD = 0.57; 95% CI: −0.48 to 1.63, *p* = 0.29, I^2^ = 56%, Figure 3).

**Figure 4:**
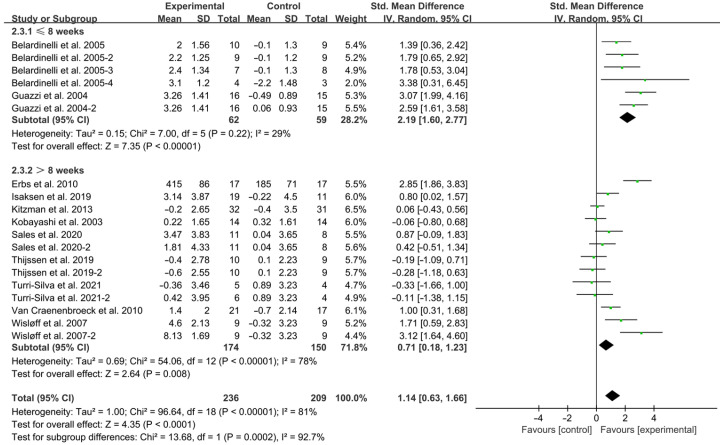
Meta-analysis results of the effects of intervention duration on FMD in HF patients [15,18,19,28,29,30,31,32,33,34,35].

Both interventions ≤ 8 weeks (SMD = 2.19; 95% CI: 1.60 to 2.77, *p* < 0.00001, I^2^ = 29%) and >8 weeks (SMD = 0.71; 95% CI: 0.18 to 1.23, *p* = 0.008, I^2^ = 78%, Figure 4) significantly improved FMD in HF patients, with shorter interventions showing greater effect sizes.

**Figure 5:**
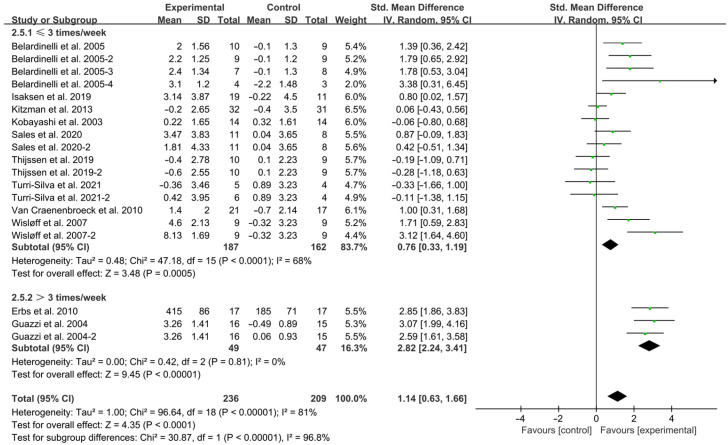
Meta-analysis results of the effects of frequency of intervention on FMD in HF patients [15,18,19,28,29,30,31,32,33,34,35].

Exercise ≤ 3 times per week (SMD = 0.76; 95% CI: 0.33 to 1.19, *p* = 0.0005, I^2^ = 68%) and >3 times per week (SMD = 2.82; 95% CI: 2.24 to 3.41, *p* < 0.00001, I^2^ = 0%, Figure 5) both significantly improved FMD in HF patients, with higher frequencies showing stronger effects.

**Figure 6:**
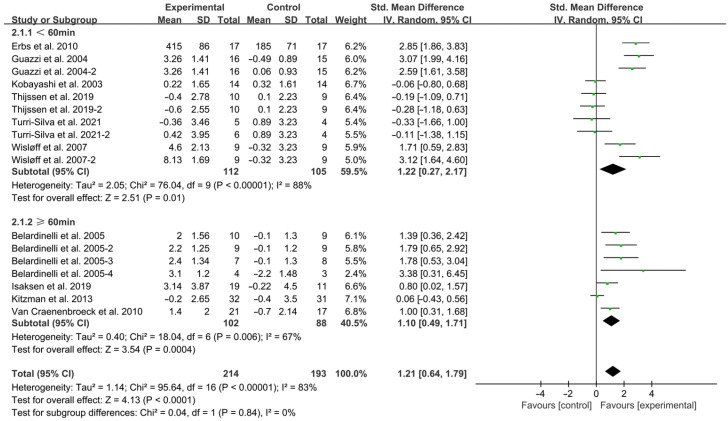
Meta-analysis results of the effects of duration of intervention per session on FMD in HF patients [15,18,19,29,30,31,32,33,34,35].

Session durations < 60 min (SMD = 1.22; 95% CI: 0.27 to 2.17, *p* = 0.01, I^2^ = 88%) and ≥60 min (SMD = 1.10; 95% CI: 0.49 to 1.71, *p* = 0.0004, I^2^ = 67%, Figure 6) improved FMD in HF patients, with shorter sessions yielding greater improvements.

**Figure 7:**
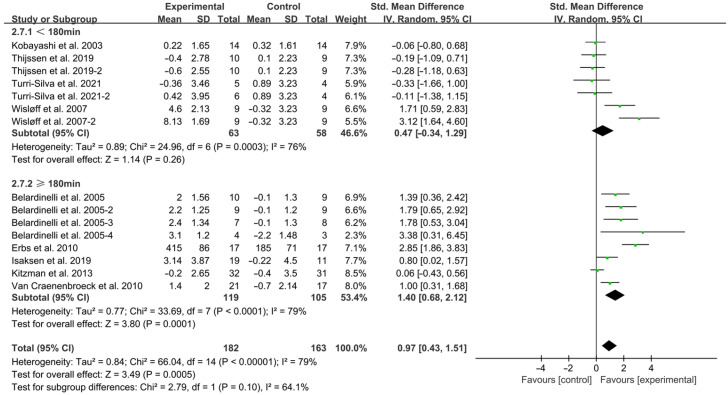
Meta-analysis results of the effects of duration of intervention per week on FMD in HF patients [15,18,19,30,31,32,33,34,35].

Exercise < 180 min per week (SMD = 0.47; 95% CI: −0.34 to 1.29, *p* = 0.0003, I^2^ = 76%) and ≥180 min per week (SMD = 1.40; 95% CI: 0.68 to 2.12, *p* < 0.0001, I^2^ = 79%, Figure 7) improved FMD in HF patients, with ≥180 min per week showing superior effects.

**Figure 8:**
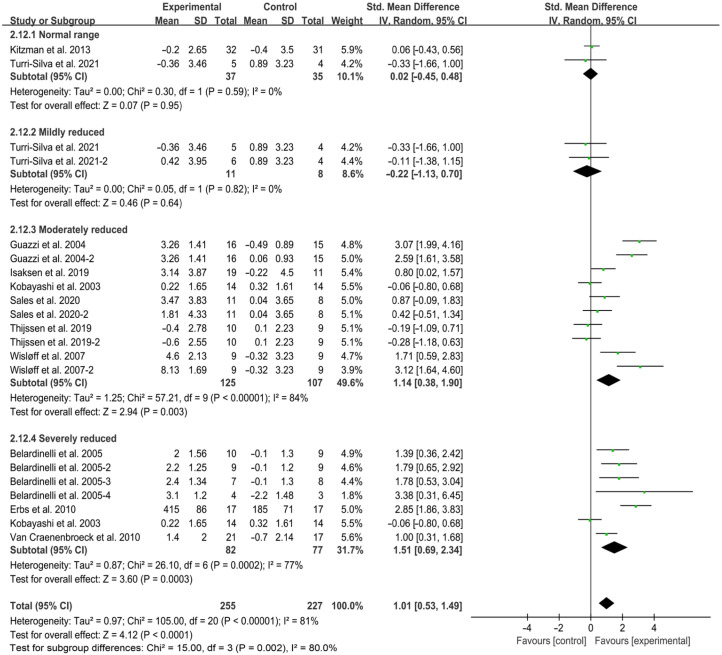
Meta-analysis of the effects of exercise on FMD in HF patients with normal range, mild, moderate, or severe endothelial impairment [15,18,19,28,29,30,31,32,33,34,35].

Exercise did not significantly improve FMD in HF patients with normal range (SMD = 0.02; 95% CI: −0.45 to 0.48, *p* = 0.95, I^2^ = 0%) or mild reduction (SMD = −0.22; 95% CI: −1.13 to 0.70, *p* = 0.64, I^2^ = 0%) in endothelial function. In contrast, significant improvements were observed in patients with moderate (SMD = 1.14; 95% CI: 0.38 to 1.90, *p* = 0.003, I^2^ = 84%) and severe (SMD = 1.51; 95% CI: 0.69 to 2.34, *p* = 0.0003, I^2^ = 77%, Figure 8) endothelial impairment.

### 3.6. Risk of Bias

Methodological quality, assessed using the RoB tool, is summarized in Figure S1. No studies were rated as low risk of bias. Seven studies were judged to have moderate risk, while four studies were classified as high risk.

### 3.7. Publication Bias

Visual inspection of the funnel plot suggested potential publication bias (Figure S2). Egger’s test confirmed this (*p* = 0.031, Table S4). Trim-and-fill analysis further indicated evidence of publication bias for FMD outcomes.

### 3.8. Sensitivity Analysis

Sensitivity analysis demonstrated that exclusion of any single study did not alter the overall findings. The beneficial effects of exercise on FMD in HF patients remained robust in both direction and magnitude (Figure S3).

### 3.9. GRADE Summary

The certainty of the evidence underpinning our findings was systematically assessed using the GRADE framework. Following this rigorous appraisal, the overall certainty of the evidence was rated as high, indicating strong confidence in the reliability and generalizability of our conclusions (Table S5).

## 4. Discussion

### 4.1. Main Findings

This study aimed to explore the effects of exercise on vascular endothelial function in HF patients, with the objective of identifying the most effective exercise modality for improving endothelial function. Our findings indicated that exercise significantly improved FMD in HF patients. Subgroup analyses further demonstrated that exercise was particularly effective in patients with severe HF, aerobic exercise had a more pronounced effect on endothelial function when the intervention duration was ≤8 weeks, the frequency exceeded 3 times per week, each session lasted < 60 min, and the total weekly exercise duration was ≥180 min.

### 4.2. The Effects of Exercise on FMD in HF Patients

Our findings indicated that exercise, particularly aerobic exercise, significantly improved FMD in HF patients, which aligns with previous studies. Belardinelli et al. [31] reported that 8 weeks of cycling at 60% VO_2_peak substantially improved brachial artery endothelial dysfunction, suggesting that lower limb exercise contributes to systemic vascular adaptation. Kobayashi et al. [32] similarly found that 12 weeks of lower-extremity cycling at 60–70% VO_2_peak significantly improved local vascular function at the training site, though no improvements were observed in untrained regions. Erbs et al. [33] demonstrated that 12 weeks of cycling at 50% VO_2_peak enhanced myocardial regenerative capacity and FMD in patients with advanced HF, while Wisløff et al. [34] showed that both aerobic interval training and moderate-intensity continuous training improved FMD, with interval training exerting greater benefits via antioxidant mechanisms and reduced oxidized low-density lipoprotein.

Delving deeper into mechanisms, previous studies have identified several pathways by which exercise may enhance FMD. First, exercise-induced shear stress promotes the synthesis and release of NO [36,37]. Periodic increases in blood flow velocity and shear stress activate mechanosensitive endothelial receptors, leading to eNOS phosphorylation and subsequent NO release [38]. NO relaxes vascular smooth muscle cells, thereby enhancing vasodilation and FMD [39]. Second, exercise reduces reactive oxygen species (ROS) production through upregulation of antioxidant enzymes [40,41]. Lower ROS levels prevent NO oxidation to peroxynitrite (ONOO^−^), thereby preserving NO-mediated vasodilation [42]. Third, exercise suppresses inflammatory responses, upregulates vascular endothelial growth factor, reduces endothelial adhesion molecule expression, and stimulates endothelial cell proliferation, collectively improving vascular permeability and FMD [43,44,45]. Thus, exercise enhances endothelial function in HF patients through multiple complementary mechanisms.

Although these findings are consistent with previous studies, some reports indicate that exercise does not significantly improve FMD in HF patients. For instance, Kitzman et al. [18] examined patients with HFpEF and found no significant FMD improvement after 16 weeks of aerobic exercise (3 times per week). Turri-Silva et al. [19] similarly observed no endothelial changes after 12 weeks of high-intensity interval training in HF patients with comorbidities such as diabetes, hypertension, or chronic kidney disease. Thijssen et al. [35] reported that 12 weeks of interval or continuous training reduced endothelial injury but did not significantly improve baseline FMD. In addition, Benda et al. [17] and Kobayashi et al. [32] noted that exercise predominantly improved endothelial function in trained regions, without systemic effects.

The lack of improvement in some studies may be attributed to several factors. First, population heterogeneity may influence outcomes; patients with HFpEF may respond less favorably to exercise than those with HFrEF, as endothelial dysfunction in HFpEF appears more resistant [46]. Second, small sample sizes may reduce statistical power [19], obscuring true effects. Third, comorbidities may exacerbate endothelial damage and attenuate exercise benefits. Fourth, insufficient exercise frequency may fail to adequately activate NO pathways and induce vascular adaptations [16]. Additionally, a ceiling effect may occur in patients with relatively high baseline FMD, limiting the potential for further improvement [35]. Finally, exercise benefits may arise primarily from local hemodynamic changes (e.g., shear stress), thereby restricting effects to trained vascular regions [17,32].

In summary, multiple confounders may limit the observed effectiveness of exercise interventions. Future studies should therefore control for potential confounding factors (e.g., diet, medication use), account for individual differences (e.g., baseline vascular function, age, comorbidities), and adopt more rigorous designs to clarify the relationship between exercise and vascular adaptation.

### 4.3. The Effects of Various Exercise Moderators on FMD in HF Patients

Among different exercise modalities, aerobic exercise demonstrated the most significant improvement in FMD in HF patients. This finding is consistent with previous studies. Deftereos et al. [47] compared aerobic exercise with FES and found that FMD improvement was more pronounced after aerobic exercise. Hambrecht et al. [38] demonstrated that six months of aerobic exercise significantly increased acetylcholine-induced blood flow in the lower extremities of HF patients, with this improvement closely associated with increased VO_2_peak. Furthermore, Green et al. [36] showed that while resistance exercise primarily induces arterial structural remodeling (e.g., diameter enlargement), its effect on endothelial function is less pronounced compared with aerobic exercise.

Aerobic exercises, such as walking and cycling, are characterized by sustained and rhythmic muscle contractions that markedly increase systemic blood flow. This generates stable and high-frequency laminar shear stress on endothelial surface, directly activating mechanosensitive receptors, leading to eNOS phosphorylation, increased NO synthesis and release, and downstream anti-inflammatory and antioxidative effects [48]. Collectively, current evidence supports aerobic exercise as an effective modality for improving FMD in HF patients. However, limited studies have investigated multicomponent training regimens, warranting further exploration in future research.

Subgroup analysis revealed that interventions lasting ≤ 8 weeks yielded greater improvements in FMD compared with those exceeding > 8 weeks. This may reflect the capacity of short-term training to induce rapid physiological adaptations. Belardinelli et al. [31] reported that 8 weeks of training (24 sessions) significantly improved myocardial perfusion and endothelial function through mechanisms involving angiogenesis and vascular adaptation. Similarly, evidence suggests that ≤ 8 weeks of high-intensity training may induce faster adaptations in oxygen uptake and vascular function [49]. By contrast, longer interventions may encounter adaptive plateaus that diminish benefits.

In addition, shorter intervention durations may enhance patient adherence [50]. Piotrowicz et al. [51] showed that an 8-week home-based remote cardiac rehabilitation program achieved 100% adherence, with significant improvements in endurance and cardiopulmonary function. Tierney et al. [52] also reported that short-term interventions (e.g., home walking programs) significantly improved exercise compliance in HF patients. Enhanced compliance, in turn, is positively correlated with improvements in endothelial function [53]. Conversely, extended interventions may result in reduced adherence due to fatigue, burnout, or external barriers, ultimately diminishing effectiveness [54]. Therefore, short-term programs (≤8 weeks) not only facilitate rapid physiological adaptation but also improve compliance, representing an efficient strategy for optimizing exercise interventions in HF patients.

Subgroup analysis indicated that exercise ≥3 times per week produced significantly greater improvements in FMD compared with <3 times per week. Myers et al. [54] reported that exercise 3–5 times per week effectively increased maximal oxygen uptake and improved cardiopulmonary function in HF patients. Similarly, Liang et al. [55] found that aerobic exercise at ≥3 times per week produced the greatest improvements in aerobic capacity and quality of life.

This may be explained by the cumulative benefits of repeated vascular stimulation. Higher-frequency programs are more likely to form behavioral habits, enhance compliance, and provide consistent hemodynamic stress to the endothelium. The American College of Sports Medicine (ACSM) and American Heart Association (AHA) recommend that older adults, including those with chronic conditions, engage in moderate-intensity exercise at least 5 days per week or vigorous-intensity exercise at least 3 days per week [56]. Kobayashi et al. [32] observed that cycling 2–3 times per week only improved lower-limb vascular function, whereas systemic improvements were limited, suggesting that higher exercise frequencies may induce broader systemic vascular adaptations. Taken together, interventions performed ≥3 times per week appear optimal for improving endothelial function in HF patients, driven by greater compliance, sustained endothelial stimulation, and systemic vascular benefits.

We also examined the optimal duration of each exercise session. Subgroup analysis revealed that sessions lasting <60 min were more effective in improving FMD compared with longer sessions. This finding aligns with AHA recommendations. Piña et al. [57] emphasized that effective exercise sessions for HF patients typically last 20–30 min, including warm-up and cool-down phases, with the total duration controlled within 60 min. Exercise within this timeframe has been shown to optimize adherence while improving endothelial function [58]. Volaklis et al. [59] similarly reported that 20–30 min of resistance exercise per session yielded optimal cardiovascular and muscle adaptations in HF patients.

Supporting this, van Tol et al. [60] demonstrated that an average session duration of approximately 50 min significantly improved both exercise capacity and endothelial function in 701 HF patients. Furthermore, Li et al. [61] reported that session durations <60 min minimized fatigue, facilitating recovery in multi-session weekly programs and sustaining long-term adherence. Physiologically, shorter sessions may maximize endothelial adaptability through efficient hemodynamic and antioxidant stimulation while avoiding excessive fatigue [34]. Thus, limiting session duration to <60 min optimizes vascular function improvements while maintaining safety and compliance.

Subgroup analysis further indicated that total weekly exercise durations ≥180 min produced significantly greater improvements in FMD compared with <180 min. Oka et al. [62] reported that 180 min of weekly walking combined with resistance exercise alleviated symptoms and improved quality of life in moderate-to-severe HF patients. Similarly, Belardinelli et al. [31] confirmed that 180 min of weekly cycling significantly improved FMD, while Erbs et al. [33] demonstrated that 180–270 min of moderate-intensity training per week optimized hemodynamic and metabolic responses without inducing overtraining.

Van Craenenbroeck et al. [15] found that 24 weeks of dynamic resistance exercise (180 min per week) significantly improved endothelial function. In line with this, the 2018 U.S. Physical Activity Guidelines [63] recommend 150–300 min per week of moderate-intensity activity for optimal health benefits. Given the need to balance cardiac tolerance with vascular adaptation, a “short but frequency” strategy is recommended: sessions lasting <60 min, ≥3 times per week, with a total of ≥180 min per week [52]. This model distributes exercise load, enhances safety, and improves compliance.

### 4.4. The Effects of Disease Severity on FMD in HF Patients

Subgroup analysis revealed that exercise more significantly improved endothelial function in patients with HFrEF compared with HFpEF. Erbs et al. [33] demonstrated that patients with advanced HF (NYHA IIIb) achieved greater improvements in cardiac and endothelial function following exercise compared with those with moderate HF (NYHA II). By contrast, Thijssen et al. [35], Kitzman et al. [18], and Turri-Silva et al. [19] reported no significant improvements in FMD among HFpEF patients after 12–16 weeks of exercise, possibly due to persistent microvascular inflammation and oxidative stress.

The HF-ACTION trial (*n* = 2331) provided further evidence, showing that aerobic exercise significantly improved health outcomes in patients with left ventricular ejection fraction (LVEF) ≤ 35% [64]. These findings suggest that patients with moderate-to-severe HF derive greater vascular benefits from exercise, likely because of their more pronounced baseline endothelial dysfunction. In contrast, HFpEF patients may exhibit a ceiling effect due to relatively preserved baseline endothelial function [32]. Therefore, exercise appears particularly effective in improving endothelial function in patients with moderate-to-severe HF and reduced LVEF [65], underscoring the need for individualized rehabilitation strategies based on disease severity.

### 4.5. Limitations

This study has several limitations. First, only 11 RCTs were included, and the overall sample size was relatively small (236 participants in the intervention group and 209 in the control group). Several studies, such as that of Turri-Silva [19], had extremely limited sample sizes (*n* = 8), which may artificially inflate effect sizes and reduce statistical power. Second, substantial heterogeneity was observed (I^2^ = 81%), which was attributable not only to variability in exercise protocols but, more importantly, to the lack of stratified analyses based on distinct HR phenotypes (e.g., reduced vs. preserved ejection fraction). Because these phenotypes differ markedly in pathophysiological mechanisms and responses to exercise, the inability to account for these differences represents a major limitation of the current evidence base. Third, endothelial function was assessed exclusively using brachial FMD, without incorporating additional vascular or inflammatory indicators such as arterial stiffness or circulating inflammatory markers. Finally, very few studies investigated resistance exercise, underscoring an important gap that warrants further research.

## 5. Conclusions

Exercise improves FMD in HF patients, with aerobic exercise emerging as the most effective modality, particularly for patients with moderate-to-severe reductions in LVEF. Based on current evidence, we recommend aerobic exercise interventions lasting no longer than 8 weeks, with a frequency of no less than 3 times per week, each session lasting less than 60 min, and a total weekly exercise duration of no less than 180 min. Such a regimen optimizes improvements in endothelial function while ensuring safety and compliance.

## Data Availability

The original contributions presented in the study are included in the article/Supplementary Materials, further inquiries can be directed to the corresponding author.

## Supplementary Materials

The following supporting information can be downloaded at: https://www.mdpi.com/article/10.3390/jcdd12120458/s1, Figure S1: Results of Cochrane risk of bias tool; Figure S2: Funnel plot; Figure S3: Sensitivity analysis results; Table S1: Search strategies; Table S2: Characteristics of the studies included in this meta-analysis; Table S3: Results of meta-regression; Table S4: Results of Egger’s test; Table S5: GRADE summary of evidence.

## Abbreviations

The following abbreviations are used in this manuscript:

HF	Heart failure
HFrEF	Heart failure with reduced ejection fraction
HFpEF	Heart failure with preserved ejection fraction
eNOS	Endothelial nitric oxide synthase
NO	Nitric oxide
FMD	Flow-mediated dilation
ACCF	American College of Cardiology Foundation
AHA	American Heart Association
CR	Cardiac rehabilitation
VO_2_peak	Peak oxygen uptake
FES	Functional electrical stimulation
IMT	Inspiratory muscle training
PRISMA	Preferred Reporting Item for Systematic Reviews and Meta-Analyses
MeSH	Medical Subject Headings
PICOS	Population, Intervention, Comparison, Outcome, Study design
RCT	Randomized controlled trial
SD	Standard deviation
RoB	Risk of Bias
SE	Standard error
ROS	Reactive oxygen species
ONOO^−^	Peroxynitrite
ACSM	American College of Sports Medicine
LEVF	Left ventricular ejection fraction
