# Supplementary material for: Effects of Exercise on Flow-Mediated Dilation in Patients with Heart Failure: A Systematic Review and Meta-Analysis of Randomized Controlled Trials
Source: J Cardiovasc Dev Dis. 2025 Nov 25;12(12):458. doi: 10.3390/jcdd12120458 (PMC12734056; doi:10.3390/jcdd12120458)
Supplement: Supplementary file 1 [file jcdd-12-00458-s001.zip › jcdd-3963487-supplementary.pdf]

Supplemental material

The effect of exercise on flow-mediated dilation in patients with heart failure: a systematic review and meta-analysis of randomized controlled trials

Figure S1. Results of Cochrane risk of bias tool.. 2
Figure S2. Funnel plot.. 3
Figure S3. Sensitivity analysis results.. 4
Table S1. Search strategies.. 5
Table S2. Characteristics of the studies included in this meta-analysis...................... 18
Table S3. Results of meta-regression.. 21
Table S4. Results of Egger's test 22
Table S5. GRADE summary of evidence...23

Figure S1. Results of Cochrane risk of bias tool.

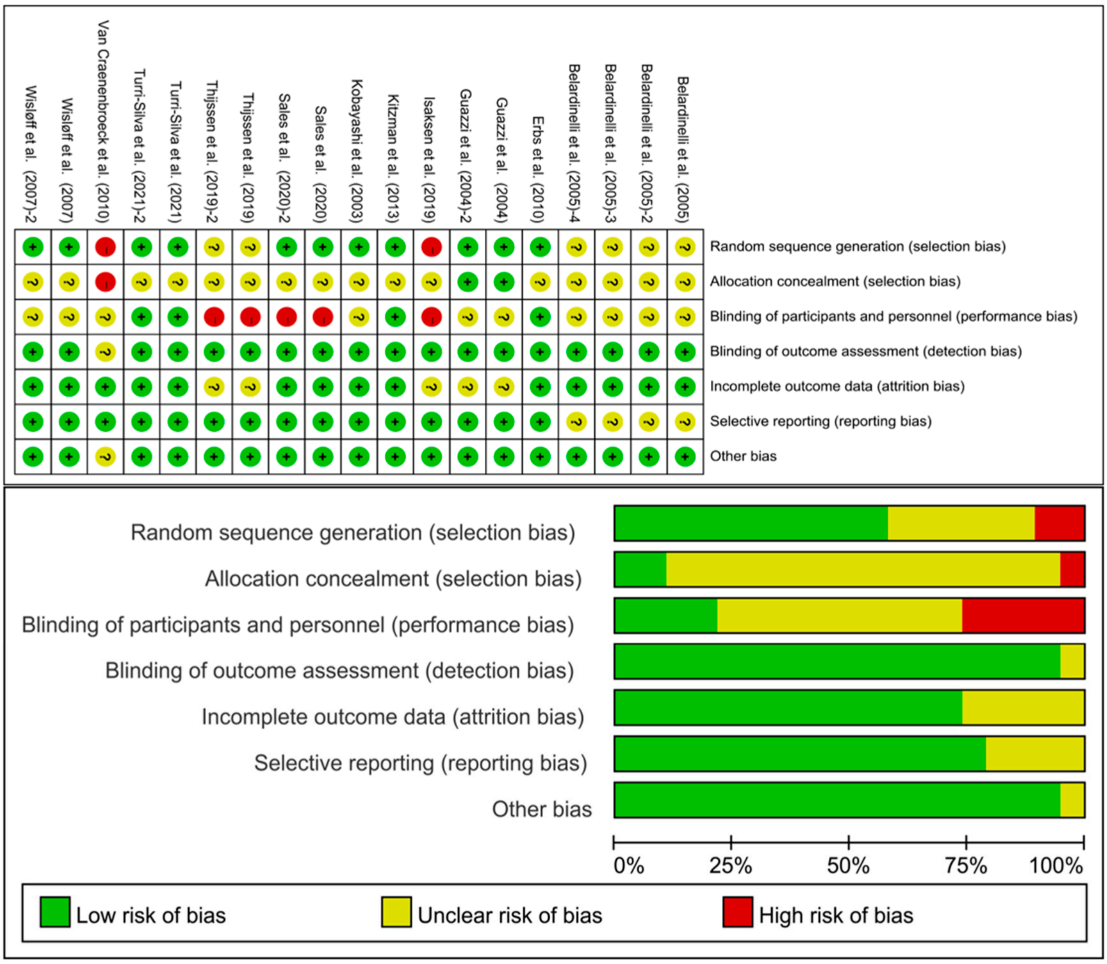

Figure S2. Funnel plot.

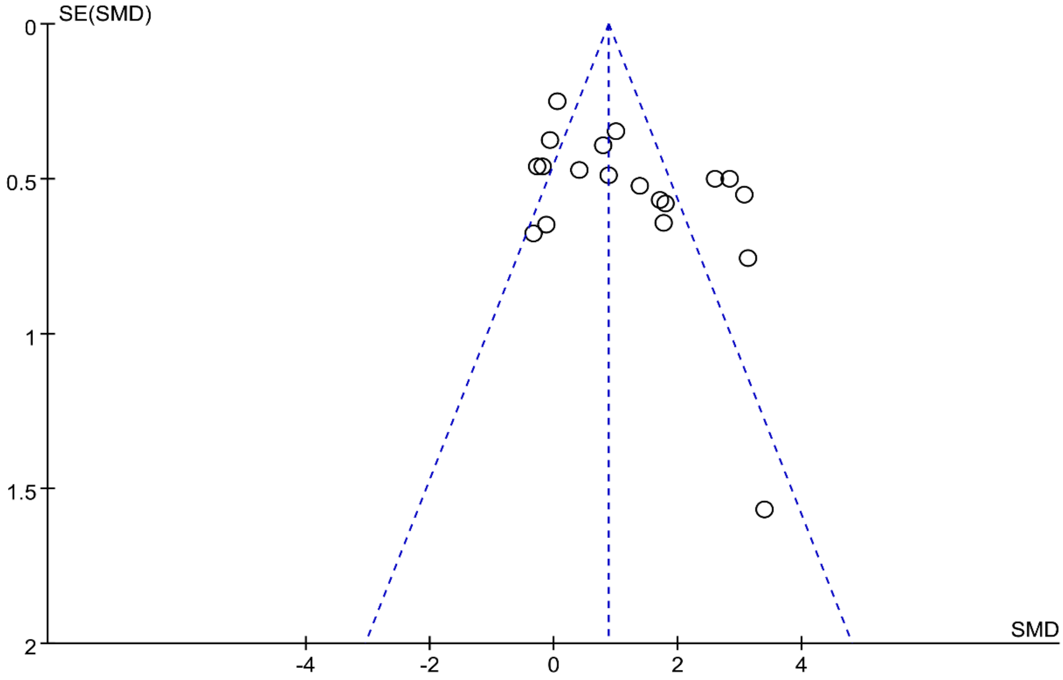

Figure S3. Sensitivity analysis results.

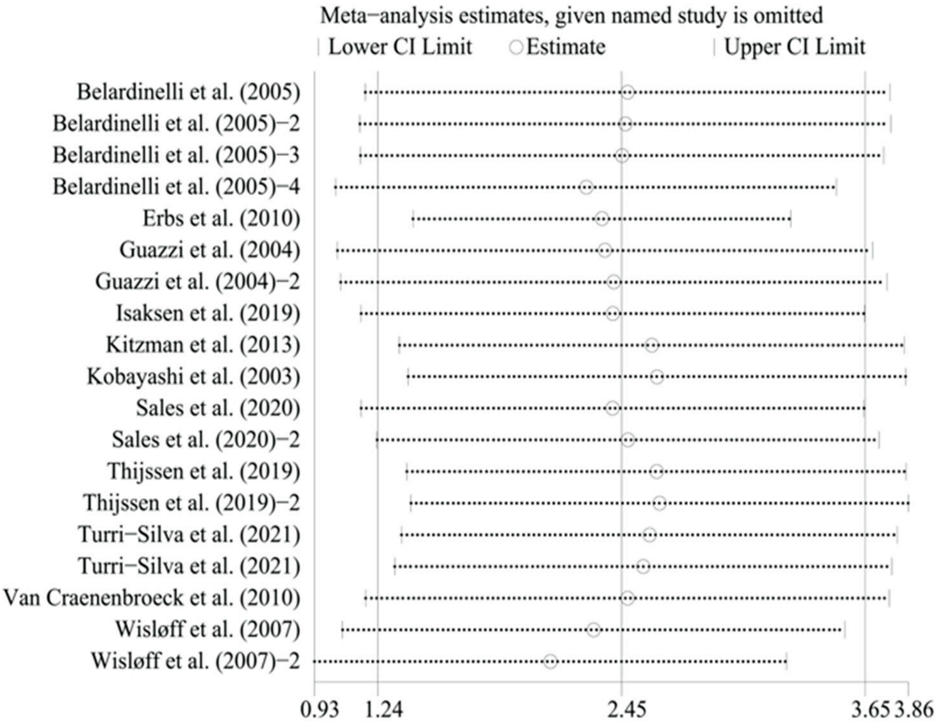

Table S1. Search strategies.

Web of Science

Term	Search	Query
Endothelium	#1	TS= ("endothelium" OR "vascular function" OR "endothelial" OR "flow-mediated dilation" OR "flow mediated dilation" OR "endothelial function" OR "FMD" OR "artery blood flow")
Exercise	#2	TS= ("Physical exercise programs" OR "Physical Therapy Modalities" OR "Physical Therapy Modalities" OR "Modalities, Physical Therapy" OR "Modality, Physical Therapy" OR "Physical Therapy Modality" OR "Physiotherapy" OR "Physiotherapies" OR "Physical Therapy Techniques" OR "Physical Therapy Technique" OR "Techniques, Physical Therapy" OR "Exercise" OR "Exercise Movement Techniques" OR "Exercise Movement Techniques" OR "Movement Techniques, Exercise" OR "Exercise Movement Technics" OR "Exercise Therapy" OR "Exercise Therapy" OR "Therapy, Exercise" OR "Exercise Therapies" OR "Therapies, Exercise" OR "Exercise, Physical" OR "Exercises, Physical" OR "Physical Exercise" OR "Physical Exercises" OR "Exercise, Isometric" OR "Exercises, Isometric" OR "Isometric Exercises" OR "Isometric Exercise" OR "Exercise, Aerobic" OR "Aerobic Exercises" OR "Aerobic Exercise" OR "Resistance Training" OR "Resistance Training" OR "Training, Resistance" OR "Strength Training" OR "Training, Strength" OR "Weight-Lifting Strengthening Program" OR "Weight-Lifting Strengthening Programs" OR "Strengthening Program, Weight-Lifting" OR "Strengthening Programs, Weight-Lifting" OR "Weight Lifting Strengthening Program" OR "Weight-Lifting Strengthening Programs" OR "Weight-Lifting Exercise Program" OR "Exercise Program, Weight-Lifting" OR "Exercise Programs, Weight-Lifting" OR "Weight Lifting Exercise Program" OR "Weight-Lifting Exercise Programs" OR "Weight-Bearing Strengthening Program" OR "Strengthening Program, Weight-Bearing" OR "Strengthening Programs, Weight-Bearing" OR "Weight Bearing Strengthening Program" OR "Weight-Bearing Strengthening Programs" OR "Weight-Bearing Exercise Program" OR "Exercise Program, Weight-Bearing" OR "Exercise Programs, Weight-Bearing" OR "Weight Bearing Exercise Program" OR "Weight-Bearing Exercise Programs")
Heart failure	#3	TS= ("Heart Failure" OR "Cardiac Failure" OR "Heart Decompensation" OR "Decompensation, Heart" OR "Heart Failure, Right-Sided" OR "Heart Failure, Right Sided" OR "Right-Sided Heart Failure" OR "Right Sided Heart Failure" OR "Myocardial Failure" OR "Congestive Heart Failure" OR "Heart Failure, Congestive" OR "Heart Failure, Left-Sided" OR "Heart Failure, Left Sided" OR "Left-Sided Heart Failure" OR "Left Sided Heart Failure")
Combined	#4	#1 AND #2 AND #3

PubMed

Term	Search	Query
Endothelium	#1	"endothelium"[MeSH Terms] OR "endothelium"[All Fields] OR "endotheliums"[All Fields] OR "endotheliums"[All Fields] OR (("blood vessels"[MeSH Terms] OR ("blood"[All Fields] AND "vessels"[All Fields]) OR "blood vessels"[All Fields] OR "vascular"[All Fields] OR "neovascularization, pathologic"[MeSH Terms] OR ("neovascularization"[All Fields] AND "pathologic"[All Fields]) OR "pathologic neovascularization"[All Fields] OR "vascularisation"[All Fields] OR "vascularization"[All Fields] OR "vascularisations"[All Fields] OR "vascularise"[All Fields] OR "vascularised"[All Fields] OR "vascularities"[All Fields] OR "vascularitis"[All Fields] OR "vascularity"[All Fields] OR "vascularizations"[All Fields] OR "vascularize"[All Fields] OR "vascularized"[All Fields] OR "vascularizes"[All Fields] OR "vascularizing"[All Fields] OR "vasculars"[All Fields]) AND ("functional"[All Fields] OR "functional s"[All Fields] OR "functionalities"[All Fields] OR "functionality"[All Fields] OR "functionalization"[All Fields] OR "functionalizations"[All Fields] OR "functionalize"[All Fields] OR "functionalized"[All Fields] OR "functionalizes"[All Fields] OR "functionalizing"[All Fields] OR "functionally"[All Fields] OR "functionals"[All Fields] OR "functioned"[All Fields] OR "functioning"[All Fields] OR "functionings"[All Fields] OR "functions"[All Fields] OR "physiology"[MeSH Subheading] OR "physiology"[All Fields] OR "function"[All Fields] OR "physiology"[MeSH Terms])) OR ("endothelialization"[All Fields] OR "endothelialize"[All Fields] OR "endothelialized"[All Fields] OR "endothelializing"[All Fields] OR "endothelials"[All Fields] OR "endothelium"[MeSH Terms] OR "endothelium"[All Fields] OR "endothelial"[All Fields]) OR ("flow-mediated"[All Fields] AND ("dilatable"[All Fields] OR "dilatated"[All Fields] OR "dilatating"[All Fields] OR "dilatation"[MeSH Terms] OR "dilatation"[All Fields] OR "dilatations"[All Fields] OR "dilate"[All Fields] OR "dilation"[All Fields] OR "dilations"[All Fields] OR "dilatative"[All Fields] OR "dilatator"[All Fields] OR "dilatators"[All Fields] OR "dilated"[All Fields] OR "dilates"[All Fields] OR "dilating"[All Fields] OR "dilator"[All Fields] OR "dilators"[All Fields])) OR (("flow camb"[Journal] OR "flow"[All Fields]) AND ("mediated"[All Fields] OR "mediational"[All Fields] OR "mediator"[All Fields] OR "mediator s"[All Fields] OR "mediators"[All Fields] OR "negotiating"[MeSH Terms] OR "negotiating"[All Fields] OR

		"mediate"[All Fields] OR "mediates"[All Fields] OR "mediating"[All Fields] OR "mediation"[All Fields] OR "mediations"[All Fields])) OR ("dilatable"[All Fields] OR "dilatated"[All Fields] OR "dilatating"[All Fields] OR "dilatation"[MeSH Terms] OR "dilatation"[All Fields] OR "dilatations"[All Fields] OR "dilate"[All Fields] OR "dilation"[All Fields] OR "dilations"[All Fields] OR "dilatative"[All Fields] OR "dilatator"[All Fields] OR "dilatators"[All Fields] OR "dilated"[All Fields] OR "dilates"[All Fields] OR "dilating"[All Fields] OR "dilator"[All Fields] OR "dilators"[All Fields]) OR (("endothelialization"[All Fields] OR "endothelialize"[All Fields] OR "endothelialized"[All Fields] OR "endothelializing"[All Fields] OR "endothelials"[All Fields] OR "endothelium"[MeSH Terms] OR "endothelium"[All Fields] OR "endothelial"[All Fields]) AND ("functional"[All Fields] OR "functional s"[All Fields] OR "functionalities"[All Fields] OR "functionality"[All Fields] OR "functionalization"[All Fields] OR "functionalizations"[All Fields] OR "functionalize"[All Fields] OR "functionalized"[All Fields] OR "functionalizes"[All Fields] OR "functionalizing"[All Fields] OR "functionally"[All Fields] OR "functionals"[All Fields] OR "functioned"[All Fields] OR "functioning"[All Fields] OR "functionings"[All Fields] OR "functions"[All Fields] OR "physiology"[MeSH Subheading] OR "physiology"[All Fields] OR "function"[All Fields] OR "physiology"[MeSH Terms])) OR "FMD"[All Fields] OR (("arterialization"[All Fields] OR "arterializations"[All Fields] OR "arterialize"[All Fields] OR "arterialized"[All Fields] OR "arterializing"[All Fields] OR "arterially"[All Fields] OR "arterials"[All Fields] OR "arterie"[All Fields] OR "arteries"[MeSH Terms] OR "arteries"[All Fields] OR "arterial"[All Fields] OR "arteris"[All Fields] OR "artery"[All Fields] OR "arterious"[All Fields] OR "artery s"[All Fields] OR "arterys"[All Fields]) AND ("blood circulation"[MeSH Terms] OR ("blood"[All Fields] AND "circulation"[All Fields]) OR "blood circulation"[All Fields] OR ("blood"[All Fields] AND "flow"[All Fields]) OR "blood flow"[All Fields]))
Exercise	#2	(("exercise"[MeSH Terms] OR "exercise"[All Fields] OR ("physical"[All Fields] AND "exercise"[All Fields]) OR "physical exercise"[All Fields]) AND ("program"[All Fields] OR "program s"[All Fields] OR "programe"[All Fields] OR "programed"[All Fields] OR "programes"[All Fields] OR "programing"[All Fields] OR "programmability"[All Fields] OR "programmable"[All Fields] OR "programmably"[All Fields] OR "programme"[All Fields] OR "programme s"[All Fields] OR "programmed"[All Fields] OR "programmer"[All Fields] OR "programmer s"[All Fields] OR

		"programmers"[All Fields] OR "programmes"[All Fields] OR "programming"[All Fields] OR "programmings"[All Fields] OR "programs"[All Fields])) OR ("physical therapy modalities"[MeSH Terms] OR ("physical"[All Fields] AND "therapy"[All Fields] AND "modalities"[All Fields]) OR "physical therapy modalities"[All Fields]) OR ("physical therapy modalities"[MeSH Terms] OR ("physical"[All Fields] AND "therapy"[All Fields] AND "modalities"[All Fields]) OR "physical therapy modalities"[All Fields]) OR ("physical therapy modalities"[MeSH Terms] OR ("physical"[All Fields] AND "therapy"[All Fields] AND "modalities"[All Fields]) OR "physical therapy modalities"[All Fields] OR ("modalities"[All Fields] AND "physical"[All Fields] AND "therapy"[All Fields]) OR "modalities physical therapy"[All Fields]) OR ("physical therapy modalities"[MeSH Terms] OR ("physical"[All Fields] AND "therapy"[All Fields] AND "modalities"[All Fields]) OR "physical therapy modalities"[All Fields] OR ("modality"[All Fields] AND "physical"[All Fields] AND "therapy"[All Fields])) OR ("physical therapy modalities"[MeSH Terms] OR ("physical"[All Fields] AND "therapy"[All Fields] AND "modalities"[All Fields]) OR "physical therapy modalities"[All Fields] OR ("physical"[All Fields] AND "therapy"[All Fields] AND "modality"[All Fields]) OR "physical therapy modality"[All Fields]) OR ("physical therapy modalities"[MeSH Terms] OR ("physical"[All Fields] AND "therapy"[All Fields] AND "modalities"[All Fields]) OR "physical therapy modalities"[All Fields] OR "physiotherapies"[All Fields] OR "physiotherapy"[All Fields]) OR ("physical therapy modalities"[MeSH Terms] OR ("physical"[All Fields] AND "therapy"[All Fields] AND "modalities"[All Fields]) OR "physical therapy modalities"[All Fields] OR "physiotherapies"[All Fields] OR "physiotherapy"[All Fields]) OR ("physical therapy modalities"[MeSH Terms] OR ("physical"[All Fields] AND "therapy"[All Fields] AND "modalities"[All Fields]) OR "physical therapy modalities"[All Fields] OR ("physical"[All Fields] AND "therapy"[All Fields] AND "techniques"[All Fields]) OR "physical therapy techniques"[All Fields]) OR ("physical therapy modalities"[MeSH Terms] OR ("physical"[All Fields] AND "therapy"[All Fields] AND "modalities"[All Fields]) OR "physical therapy modalities"[All Fields] OR ("physical"[All Fields] AND "therapy"[All Fields]) OR "physical therapy"[All Fields]) OR ("methods"[MeSH Terms] OR "methods"[All Fields] OR "technique"[All Fields] OR "methods"[MeSH Subheading] OR "techniques"[All Fields] OR "technique s"[All Fields]) OR ("physical therapy modalities"[MeSH Terms] OR ("physical"[All Fields] AND "therapy"[All Fields] AND "modalities"[All Fields]) OR "physical

		therapy modalities"[All Fields] OR ("techniques"[All Fields] AND "physical"[All Fields] AND "therapy"[All Fields]) OR "techniques physical therapy"[All Fields]) OR ("exercise"[MeSH Terms] OR "exercise"[All Fields] OR "exercises"[All Fields] OR "exercise therapy"[MeSH Terms] OR ("exercise"[All Fields] AND "therapy"[All Fields]) OR "exercise therapy"[All Fields] OR "exercising"[All Fields] OR "exercise s"[All Fields] OR "exercised"[All Fields] OR "exerciser"[All Fields] OR "exercisers"[All Fields]) OR ("exercise movement techniques"[MeSH Terms] OR ("exercise"[All Fields] AND "movement"[All Fields] AND "techniques"[All Fields]) OR "exercise movement techniques"[All Fields]) OR ("exercise movement techniques"[MeSH Terms] OR ("exercise"[All Fields] AND "movement"[All Fields] AND "techniques"[All Fields]) OR "exercise movement techniques"[All Fields]) OR ("exercise movement techniques"[MeSH Terms] OR ("exercise"[All Fields] AND "movement"[All Fields] AND "techniques"[All Fields]) OR "exercise movement techniques"[All Fields] OR ("movement"[All Fields] AND "techniques"[All Fields] AND "exercise"[All Fields])) OR ("exercise movement techniques"[MeSH Terms] OR ("exercise"[All Fields] AND "movement"[All Fields] AND "techniques"[All Fields]) OR "exercise movement techniques"[All Fields] OR ("exercise"[All Fields] AND "movement"[All Fields] AND "technics"[All Fields]) OR "exercise movement technics"[All Fields]) OR ("exercise therapy"[MeSH Terms] OR ("exercise"[All Fields] AND "therapy"[All Fields]) OR "exercise therapy"[All Fields]) OR ("exercise therapy"[MeSH Terms] OR ("exercise"[All Fields] AND "therapy"[All Fields]) OR "exercise therapy"[All Fields]) OR ("exercise therapy"[MeSH Terms] OR ("exercise"[All Fields] AND "therapy"[All Fields]) OR "exercise therapy"[All Fields] OR ("therapy"[All Fields] AND "exercise"[All Fields]) OR "therapy exercise"[All Fields]) OR ("exercise therapy"[MeSH Terms] OR ("exercise"[All Fields] AND "therapy"[All Fields]) OR "exercise therapy"[All Fields] OR ("exercise"[All Fields] AND "therapies"[All Fields]) OR "exercise therapies"[All Fields]) OR ("exercise therapy"[MeSH Terms] OR ("exercise"[All Fields] AND "therapy"[All Fields]) OR "exercise therapy"[All Fields] OR ("therapies"[All Fields] AND "exercise"[All Fields]) OR "therapies exercise"[All Fields]) OR ("exercise"[MeSH Terms] OR "exercise"[All Fields] OR ("exercise"[All Fields] AND "physical"[All Fields]) OR "exercise physical"[All Fields]) OR ("exercise"[MeSH Terms] OR "exercise"[All Fields] OR ("exercises"[All Fields] AND "physical"[All Fields]) OR "exercises physical"[All Fields]) OR ("exercise"[MeSH Terms] OR

		"exercise"[All Fields] OR ("physical"[All Fields] AND "exercise"[All Fields]) OR "physical exercise"[All Fields]) OR ("exercise"[MeSH Terms] OR "exercise"[All Fields] OR ("physical"[All Fields] AND "exercises"[All Fields]) OR "physical exercises"[All Fields]) OR ("exercise"[MeSH Terms] OR "exercise"[All Fields] OR ("exercise"[All Fields] AND "isometric"[All Fields]) OR "exercise isometric"[All Fields]) OR ("exercise"[MeSH Terms] OR "exercise"[All Fields] OR ("exercises"[All Fields] AND "isometric"[All Fields]) OR "exercises isometric"[All Fields]) OR ("exercise"[MeSH Terms] OR "exercise"[All Fields] OR ("isometric"[All Fields] AND "exercises"[All Fields]) OR "isometric exercises"[All Fields]) OR ("exercise"[MeSH Terms] OR "exercise"[All Fields] OR ("isometric"[All Fields] AND "exercise"[All Fields]) OR "isometric exercise"[All Fields]) OR ("exercise"[MeSH Terms] OR "exercise"[All Fields] OR ("exercise"[All Fields] AND "aerobic"[All Fields]) OR "exercise aerobic"[All Fields]) OR ("exercise"[MeSH Terms] OR "exercise"[All Fields] OR ("aerobic"[All Fields] AND "exercises"[All Fields]) OR "aerobic exercises"[All Fields]) OR ("exercise"[MeSH Terms] OR "exercise"[All Fields] OR ("aerobic"[All Fields] AND "exercise"[All Fields]) OR "aerobic exercise"[All Fields]) OR ("resistance training"[MeSH Terms] OR ("resistance"[All Fields] AND "training"[All Fields]) OR "resistance training"[All Fields]) OR ("resistance training"[MeSH Terms] OR ("resistance"[All Fields] AND "training"[All Fields]) OR "resistance training"[All Fields]) OR ("resistance training"[MeSH Terms] OR ("resistance"[All Fields] AND "training"[All Fields]) OR "resistance training"[All Fields] OR ("training"[All Fields] AND "resistance"[All Fields]) OR "training resistance"[All Fields]) OR ("resistance training"[MeSH Terms] OR ("resistance"[All Fields] AND "training"[All Fields]) OR "resistance training"[All Fields] OR ("strength"[All Fields] AND "training"[All Fields]) OR "strength training"[All Fields]) OR ("resistance training"[MeSH Terms] OR ("resistance"[All Fields] AND "training"[All Fields]) OR "resistance training"[All Fields] OR ("training"[All Fields] AND "strength"[All Fields]) OR "training strength"[All Fields]) OR ("resistance training"[MeSH Terms] OR ("resistance"[All Fields] AND "training"[All Fields]) OR "resistance training"[All Fields] OR ("weight"[All Fields] AND "lifting"[All Fields] AND "strengthening"[All Fields] AND "program"[All Fields])) OR ("resistance training"[MeSH Terms] OR ("resistance"[All Fields] AND "training"[All Fields]) OR "resistance training"[All Fields] OR ("strengthening"[All Fields] AND "program"[All Fields] AND "weight"[All Fields] AND "lifting"[All Fields])) OR ("resistance

		training"[MeSH Terms] OR ("resistance"[All Fields] AND "training"[All Fields]) OR "resistance training"[All Fields] OR ("strengthening"[All Fields] AND "programs"[All Fields] AND "weight"[All Fields] AND "lifting"[All Fields])) OR ("resistance training"[MeSH Terms] OR ("resistance"[All Fields] AND "training"[All Fields]) OR "resistance training"[All Fields] OR ("weight"[All Fields] AND "lifting"[All Fields] AND "strengthening"[All Fields] AND "program"[All Fields])) OR ("resistance training"[MeSH Terms] OR ("resistance"[All Fields] AND "training"[All Fields]) OR "resistance training"[All Fields] OR ("weight"[All Fields] AND "lifting"[All Fields] AND "strengthening"[All Fields] AND "programs"[All Fields])) OR ("resistance training"[MeSH Terms] OR ("resistance"[All Fields] AND "training"[All Fields]) OR "resistance training"[All Fields] OR ("weight"[All Fields] AND "lifting"[All Fields] AND "exercise"[All Fields] AND "program"[All Fields]) OR "weight lifting exercise program"[All Fields]) OR ("resistance training"[MeSH Terms] OR ("resistance"[All Fields] AND "training"[All Fields]) OR "resistance training"[All Fields] OR ("exercise"[All Fields] AND "program"[All Fields] AND "weight"[All Fields] AND "lifting"[All Fields])) OR ("resistance training"[MeSH Terms] OR ("resistance"[All Fields] AND "training"[All Fields]) OR "resistance training"[All Fields] OR ("exercise"[All Fields] AND "programs"[All Fields] AND "weight"[All Fields] AND "lifting"[All Fields])) OR ("resistance training"[MeSH Terms] OR ("resistance"[All Fields] AND "training"[All Fields]) OR "resistance training"[All Fields] OR ("weight"[All Fields] AND "lifting"[All Fields] AND "exercise"[All Fields] AND "program"[All Fields]) OR "weight lifting exercise program"[All Fields]) OR ("resistance training"[MeSH Terms] OR ("resistance"[All Fields] AND "training"[All Fields]) OR "resistance training"[All Fields] OR ("weight"[All Fields] AND "lifting"[All Fields] AND "exercise"[All Fields] AND "programs"[All Fields]) OR "weight lifting exercise programs"[All Fields]) OR ("resistance training"[MeSH Terms] OR ("resistance"[All Fields] AND "training"[All Fields]) OR "resistance training"[All Fields] OR ("weight"[All Fields] AND "bearing"[All Fields] AND "strengthening"[All Fields] AND "program"[All Fields]) OR "weight bearing strengthening program"[All Fields]) OR ("resistance training"[MeSH Terms] OR ("resistance"[All Fields] AND "training"[All Fields]) OR "resistance training"[All Fields] OR ("strengthening"[All Fields] AND "program"[All Fields] AND "weight"[All Fields] AND "bearing"[All Fields])) OR ("resistance training"[MeSH Terms] OR ("resistance"[All Fields] AND "training"[All Fields]) OR "resistance training"[All Fields] OR

		("strengthening"[All Fields] AND "programs"[All Fields] AND "weight"[All Fields] AND "bearing"[All Fields]) OR "strengthening programs weight bearing"[All Fields]) OR ("resistance training"[MeSH Terms] OR ("resistance"[All Fields] AND "training"[All Fields]) OR "resistance training"[All Fields] OR ("weight"[All Fields] AND "bearing"[All Fields] AND "strengthening"[All Fields] AND "program"[All Fields]) OR "weight bearing strengthening program"[All Fields]) OR ("resistance training"[MeSH Terms] OR ("resistance"[All Fields] AND "training"[All Fields]) OR "resistance training"[All Fields] OR ("weight"[All Fields] AND "bearing"[All Fields] AND "strengthening"[All Fields] AND "programs"[All Fields]) OR "weight bearing strengthening programs"[All Fields]) OR ("resistance training"[MeSH Terms] OR ("resistance"[All Fields] AND "training"[All Fields]) OR "resistance training"[All Fields] OR ("weight"[All Fields] AND "bearing"[All Fields] AND "exercise"[All Fields] AND "program"[All Fields]) OR "weight bearing exercise program"[All Fields]) OR ("resistance training"[MeSH Terms] OR ("resistance"[All Fields] AND "training"[All Fields]) OR "resistance training"[All Fields] OR ("exercise"[All Fields] AND "program"[All Fields] AND "weight"[All Fields] AND "bearing"[All Fields])) OR ("resistance training"[MeSH Terms] OR ("resistance"[All Fields] AND "training"[All Fields]) OR "resistance training"[All Fields] OR ("exercise"[All Fields] AND "programs"[All Fields] AND "weight"[All Fields] AND "bearing"[All Fields]) OR "exercise programs weight bearing"[All Fields]) OR ("resistance training"[MeSH Terms] OR ("resistance"[All Fields] AND "training"[All Fields]) OR "resistance training"[All Fields] OR ("weight"[All Fields] AND "bearing"[All Fields] AND "exercise"[All Fields] AND "program"[All Fields]) OR "weight bearing exercise program"[All Fields]) OR ("resistance training"[MeSH Terms] OR ("resistance"[All Fields] AND "training"[All Fields]) OR "resistance training"[All Fields] OR ("weight"[All Fields] AND "bearing"[All Fields] AND "exercise"[All Fields] AND "programs"[All Fields]) OR "weight bearing exercise programs"[All Fields])
Heart failure		"heart failure"[MeSH Terms] OR ("heart"[All Fields] AND "failure"[All Fields]) OR "heart failure"[All Fields] OR ("heart failure"[MeSH Terms] OR ("heart"[All Fields] AND "failure"[All Fields]) OR "heart failure"[All Fields] OR ("cardiac"[All Fields] AND "failure"[All Fields]) OR "cardiac failure"[All Fields]) OR ("heart failure"[MeSH Terms] OR ("heart"[All Fields] AND "failure"[All Fields]) OR "heart failure"[All Fields] OR ("heart"[All Fields] AND "decompensation"[All Fields]) OR "heart decompensation"[All Fields]) OR ("heart failure"[MeSH Terms] OR

	#3	("heart"[All Fields] AND "failure"[All Fields]) OR "heart failure"[All Fields] OR ("decompensation"[All Fields] AND "heart"[All Fields]) OR "decompensation heart"[All Fields]) OR ("heart failure"[MeSH Terms] OR ("heart"[All Fields] AND "failure"[All Fields]) OR "heart failure"[All Fields] OR ("heart"[All Fields] AND "failure"[All Fields] AND "right"[All Fields] AND "sided"[All Fields]) OR "heart failure right sided"[All Fields]) OR ("heart failure"[MeSH Terms] OR ("heart"[All Fields] AND "failure"[All Fields]) OR "heart failure"[All Fields] OR ("heart"[All Fields] AND "failure"[All Fields] AND "right"[All Fields] AND "sided"[All Fields]) OR "heart failure right sided"[All Fields]) OR ("heart failure"[MeSH Terms] OR ("heart"[All Fields] AND "failure"[All Fields]) OR "heart failure"[All Fields] OR ("right"[All Fields] AND "sided"[All Fields] AND "heart"[All Fields] AND "failure"[All Fields]) OR "right sided heart failure"[All Fields]) OR ("heart failure"[MeSH Terms] OR ("heart"[All Fields] AND "failure"[All Fields]) OR "heart failure"[All Fields] OR ("right"[All Fields] AND "sided"[All Fields] AND "heart"[All Fields] AND "failure"[All Fields]) OR "right sided heart failure"[All Fields]) OR ("heart failure"[MeSH Terms] OR ("heart"[All Fields] AND "failure"[All Fields]) OR "heart failure"[All Fields] OR ("myocardial"[All Fields] AND "failure"[All Fields]) OR "myocardial failure"[All Fields]) OR ("heart failure"[MeSH Terms] OR ("heart"[All Fields] AND "failure"[All Fields]) OR "heart failure"[All Fields] OR ("congestive"[All Fields] AND "heart"[All Fields] AND "failure"[All Fields]) OR "congestive heart failure"[All Fields]) OR ("heart failure"[MeSH Terms] OR ("heart"[All Fields] AND "failure"[All Fields]) OR "heart failure"[All Fields] OR ("heart"[All Fields] AND "failure"[All Fields] AND "congestive"[All Fields]) OR "heart failure congestive"[All Fields]) OR ("heart failure"[MeSH Terms] OR ("heart"[All Fields] AND "failure"[All Fields]) OR "heart failure"[All Fields] OR ("heart"[All Fields] AND "failure"[All Fields] AND "left"[All Fields] AND "sided"[All Fields]) OR "heart failure left sided"[All Fields]) OR ("heart failure"[MeSH Terms] OR ("heart"[All Fields] AND "failure"[All Fields]) OR "heart failure"[All Fields] OR ("heart"[All Fields] AND "failure"[All Fields] AND "left"[All Fields] AND "sided"[All Fields]) OR "heart failure left sided"[All Fields]) OR ("heart failure"[MeSH Terms] OR ("heart"[All Fields] AND "failure"[All Fields]) OR "heart failure"[All Fields] OR ("left"[All Fields] AND "sided"[All Fields] AND "heart"[All Fields] AND "failure"[All Fields]) OR "left sided heart failure"[All Fields]) OR ("heart failure"[MeSH Terms] OR ("heart"[All Fields] AND "failure"[All Fields]) OR "heart failure"[All Fields] OR ("left"[All Fields] AND "sided"[All Fields] AND "heart"[All Fields] AND "failure"[All Fields]) OR "left sided heart

		failure"[All Fields])
Combined	#4	#1 AND #2 AND #3

Cochrane Library

Term	Search	Query
Endothelium	#1	(Endothelium OR vascular function OR endothelial OR flow-mediated dilation OR flow mediated OR dilation OR endothelial function OR FMD OR artery blood flow):ti,ab,kw
Exercise	#2	(Physical exercise programs OR Physical Therapy Modalities OR Physical Therapy Modalities OR Modalities, Physical Therapy OR Modality, Physical Therapy OR Physical Therapy Modality OR Physiotherapy OR Physiotherapies OR Physical Therapy Techniques OR Physical Therapy OR Technique OR Techniques, Physical Therapy OR Exercise OR Exercise Movement Techniques OR Exercise Movement Techniques OR Movement Techniques, Exercise OR Exercise Movement Technics OR Exercise Therapy OR Exercise Therapy OR Therapy, Exercise OR Exercise Therapies Therapies, Exercise OR Exercise, Physical OR Exercises, Physical OR Physical Exercise OR Physical Exercises OR Exercise, Isometric OR Exercises, Isometric OR Isometric Exercises OR Isometric Exercise OR Exercise, Aerobic OR Aerobic Exercises OR Aerobic Exercise OR Resistance Training OR Resistance Training OR Training, Resistance OR Strength Training OR Training, Strength OR Weight-Lifting Strengthening Program OR Strengthening Program, Weight-Lifting OR Strengthening Programs, Weight-Lifting OR Weight Lifting Strengthening Program OR Weight-Lifting Strengthening Programs OR Weight-Lifting Exercise Program OR Exercise Program, Weight-Lifting OR Exercise Programs, Weight-Lifting OR Weight Lifting Exercise Program OR Weight-Lifting Exercise Programs OR Weight-Bearing Strengthening Program OR Strengthening Program, Weight-Bearing OR Strengthening Programs, Weight-Bearing OR Weight Bearing Strengthening Program OR Weight-Bearing Strengthening Programs OR Weight-Bearing Exercise Program OR Exercise Program, Weight-Bearing OR Exercise Programs, Weight-Bearing Weight Bearing Exercise Program OR Weight-Bearing Exercise Programs):ti,ab,kw
Heart failure	#3	(Heart Failure OR Cardiac Failure OR Heart Decompensation OR Decompensation, Heart OR Heart Failure, Right-Sided OR Heart Failure, Right Sided OR Right-Sided Heart Failure OR Right Sided Heart Failure OR Myocardial Failure OR Congestive Heart Failure OR Heart Failure, Congestive Heart Failure, Left-Sided OR Heart Failure, Left Sided OR Left-Sided Heart Failure OR Left Sided Heart Failure):ti,ab,kw

Combined	#4	#1 AND #2 AND #3

Embase

Term	Search	Query
Endothelium	#1	'endothelium:ab,ti OR 'vascular function':ab,ti OR endothelial:ab,ti OR 'flow-mediated dilation':ab,ti OR 'flow mediated':ab,ti OR 'dilation':ab,ti OR 'endothelial function':ab,ti OR 'fmd':ab,ti OR 'artery blood flow':ab,ti
Exercise	#2	'physical exercise programs':ab,ti OR 'physical therapy modalities':ab,ti OR 'modalities, physical therapy':ab,ti OR 'modality, physical therapy':ab,ti OR 'physical therapy modality':ab,ti OR physiotherapy:ab,ti OR physiotherapies:ab,ti OR 'physical therapy techniques':ab,ti OR 'physical therapy':ab,ti OR technique:ab,ti OR 'techniques, physical therapy':ab,ti OR exercise:ab,ti OR 'exercise movement techniques':ab,ti OR 'movement techniques, exercise':ab,ti OR 'exercise movement technics':ab,ti OR 'exercise therapy':ab,ti OR 'therapy, exercise':ab,ti OR 'exercise therapies therapies, exercise':ab,ti OR 'exercise, physical':ab,ti OR 'exercises, physical':ab,ti OR 'physical exercise':ab,ti OR 'physical exercises':ab,ti OR 'exercise, isometric':ab,ti OR 'exercises, isometric':ab,ti OR 'isometric exercises':ab,ti OR 'isometric exercise':ab,ti OR 'exercise, aerobic':ab,ti OR 'aerobic exercises':ab,ti OR 'aerobic exercise':ab,ti OR 'resistance training':ab,ti OR 'training, resistance':ab,ti OR 'strength training':ab,ti OR 'training, strength':ab,ti OR 'weight-lifting strengthening program':ab,ti OR 'strengthening program, weight-lifting':ab,ti OR 'strengthening programs, weight-lifting':ab,ti OR 'weight lifting strengthening program':ab,ti OR 'weight-lifting strengthening programs':ab,ti OR 'weight-lifting exercise program':ab,ti OR 'exercise program, weight-lifting':ab,ti OR 'exercise programs, weight-lifting':ab,ti OR 'weight lifting exercise program':ab,ti OR 'weight-lifting exercise programs':ab,ti OR 'weight-bearing strengthening program':ab,ti OR 'strengthening program, weight-bearing':ab,ti OR 'strengthening programs, weight-bearing':ab,ti OR 'weight bearing strengthening program':ab,ti OR 'weight-bearing strengthening programs':ab,ti OR 'weight-bearing exercise program':ab,ti OR 'exercise program, weight-bearing':ab,ti OR 'exercise programs, weight-bearing weight bearing exercise program':ab,ti OR 'weight-bearing exercise programs':ab,ti
Heart failure	#3	'heart failure':ab,ti OR 'cardiac failure':ab,ti OR 'heart decompensation':ab,ti OR 'decompensation, heart':ab,ti OR 'heart failure, right-sided':ab,ti OR 'heart failure, right sided':ab,ti OR 'right-sided heart failure':ab,ti OR 'right sided heart failure':ab,ti OR

		'myocardial failure':ab,ti OR 'congestive heart failure':ab,ti OR 'heart failure, congestive heart failure, left-sided':ab,ti OR 'heart failure, left sided':ab,ti OR 'left-sided heart failure':ab,ti OR 'left sided heart failure':ab,ti
Combined	#4	#1 AND #2 AND #3

Scopus

Term	Search	Query
Endothelium	#1	TITLE-ABS-KEY("endothelium" OR "vascular function" OR "endothelial" OR "flow-mediated dilation" OR "flow mediated dilation" OR "endothelial function" OR "FMD" OR "artery blood flow")
Exercise	#2	TITLE-ABS-KEY("Physical exercise programs" OR "Physical Therapy Modalities" OR "Physical Therapy Modalities" OR "Modalities, Physical Therapy" OR "Modality, Physical Therapy" OR "Physical Therapy Modality" OR "Physiotherapy" OR "Physiotherapies" OR "Physical Therapy Techniques" OR "Physical Therapy Technique" OR "Techniques, Physical Therapy" OR "Exercise" OR "Exercise Movement Techniques" OR "Exercise Movement Techniques" OR "Movement Techniques, Exercise" OR "Exercise Movement Technics" OR "Exercise Therapy" OR "Exercise Therapy" OR "Therapy, Exercise" OR "Exercise Therapies" OR "Therapies, Exercise" OR "Exercise, Physical" OR "Exercises, Physical" OR "Physical Exercise" OR "Physical Exercises" OR "Exercise, Isometric" OR "Exercises, Isometric" OR "Isometric Exercises" OR "Isometric Exercise" OR "Exercise, Aerobic" OR "Aerobic Exercises" OR "Aerobic Exercise" OR "Resistance Training" OR "Resistance Training" OR "Training, Resistance" OR "Strength Training" OR "Training, Strength" OR "Weight-Lifting Strengthening Program" OR "Strengthening Program, Weight-Lifting" OR "Strengthening Programs, Weight-Lifting" OR "Weight Lifting Strengthening Program" OR "Weight-Lifting Strengthening Programs" OR "Weight-Lifting Exercise Program" OR "Exercise Program, Weight-Lifting" OR "Exercise Programs, Weight-Lifting" OR "Weight Lifting Exercise Program" OR "Weight-Lifting Exercise Programs" OR "Weight-Bearing Strengthening Program" OR "Weight-Bearing Strengthening Programs" OR "Weight-Bearing Exercise Program" OR "Exercise Program, Weight-Bearing" OR "Exercise Programs, Weight-Bearing" OR "Weight Bearing Exercise Program" OR "Weight-Bearing Exercise Programs")
Heart failure	#3	TITLE-ABS-KEY("Heart Failure" OR "Cardiac Failure" OR "H eart Decompensation" OR "Decompensation, Heart" OR "Heart

		Failure, Right-Sided" OR "Heart Failure, Right Sided" OR "Right-Sided Heart Failure" OR "Right Sided Heart Failure" OR " Myocardial Failure" OR "Congestive Heart Failure" OR "Heart Failure, Congestive" OR "Heart Failure, Left-Sided" OR "Heart Failure, Left Sided" OR "Left-Sided Heart Failure" OR "Left Sided Heart Failure")
Combined	#4	#1 AND #2 AND #3

Table S2. Characteristics of the studies included in this meta-analysis.

Study	Sample size	Age (y)	Stage of disease	Intervention	Characteristics of intervention
Van Craenenbroeck et al. 2010	TG = 21<br>CG = 17	TG: 61.3 (2.2)<br>CG: 63.4 (3)	NYHA class II/III%	TG: endurance training<br>CG: non-trained	24 weeks; 3 times/wk; 60min/session; endurance training intensity: 90% HR.
Turri-Silva et al. (2021)	HIIT = 8<br>CRT = 6<br>CG = 8	HIIT: 60.9 (9.7)<br>CRT: 55.0 (10.9)<br>CG: 56.0 (9.7)	NYHA class I/II%	HIIT: high-intensity interval training<br>CRT: circuit-resistance training<br>CG: keep routine	HIIT: 36 sessions; 3 times/wk; 50min/session; received high-intensity interval training<br>CRT: 36 sessions; 3 times/wk; 50min/session; received circuit-resistance training
Kobayashi et al. (2003)	TG = 14<br>CG = 14	TG: 55 (2)<br>CG: 62 (2)	NYHA class II/III%	TG: aerobic exercise<br>CG: usual care	12 weeks; 2-3 days/wk; 2 sessions/day; 15min/session; underwent supervised cycle ergometer training
Kitzman et al. (2013)	TG = 32<br>CG = 31	TG: 70 (7)<br>CG: 70 (7)	NYHA class II/III%	TG: endurance training<br>CG: usual care	16 weeks; 3 times/wk; 1h/session; initially at 40%-50% HR to 70% HR
Erbs et al. (2010)	TG = 18<br>CG = 19	TG: 60 (11)<br>CG: 62 (10)	NYHA class III%	TG: aerobic exercise<br>CG: non-trained	12 weeks; 3 to 6 times daily for the first 3 weeks; 5 to 20 minutes on a bicycle ergometer for the initial phase; exercise daily for 20 to 30 minutes; 50% of VO_2max initially, with a target heart rate of 60% VO_2max for home training.
Belardinelli et al. (2005)	TG = 30<br>CG = 29	TG: 55.9 (15)<br>CG: 58 (12)	NYHA class II/III%	TG: aerobic exercise<br>CG: usual care	

					8 weeks; 3 times/wk; 60min/session; underwent supervised cycle ergometer ET at 60% VO_2peak.
Wisløff et al. (2007)	MCT = 9<br>AIT = 9<br>CG = 9	MCT: 74.4 (12)<br>AIT: 76.5 (9)<br>CG: 75.5 (13)	NYHA class II/III%	MCT: moderate continuous training<br>AIT: aerobic interval training<br>CG: supervised training once every 3 weeks	MCT: 12 weeks; 3 times/wk:walked continuously at 70% to 75% of peak heart rate for 47 minutes each session.<br>AIT: warmed up for 10 minutes at 50% to 60% of VO_2peak before walking four 4-minute intervals at 90% to 95% of peak heart rate
Sales et al. (2020)	HIIT = 11<br>MICT = 11<br>CG = 8	NR	NYHA class II/III%	HIIT: high-Intensity interval training<br>MICT: moderate-intensity continuous training<br>CG: non-trained	HIIT: 12weeks; 3 times/wk; based on the HR, corresponding to 5% above the respiratory compensation point obtained in cardiopulmonary exercise test.<br>MICT: 12week; 3 times/wk; based on the HR between anaerobic threshold and respiratory compensation point.
Guazzi et al. (2004)	TG = 16<br>CG = 15	TG: 52 (5)<br>CG: 54 (4)	NYHA class II/III%	TG: aerobic exercise<br>CG: maintain their normal daily activity	8 weeks; 4 time/wk; completed a 5-min warm-up phase, a 30-min aerobic phase, and a 5-min cool-down phase, exercise intensity was set at 60% for the first 2 wk and then increased, as tolerated, to as high as 80%.
Isaksen et al. (2019)	TG = 19<br>CG = 11	TG: 66 (9)<br>CG: 69 (9)	NYHA class I/II%	TG: aerobic exercise<br>CG: maintained usual lifestyle	12 weeks; 3 times/wk; 60min/time; patients then performed four 4-minute intervals at 85% of maximal HR.

Thijssen et al. (2019)	HIT = 10<br>CT = 10<br>CG = 9	HIT: 63 (8)<br>CT: 64 (8)<br>CG: 67 (7)	NYHA class II/III%	HIT: high-intensity training<br>CT: continuous training<br>CG: daily physical activities	HIT: 12 weeks; consisted of 10 periods of intervals of 1-min at 90% Wmax followed by 2.5-min at 30% Wmax, aiming at a Borg score of 15–17 during the high-intensity intervals.<br>CT: 12 weeks; consisted of 30-min at 60-75% Wmax, aiming at a Borg score of 12-14.

Abbreviations: HF, Heart Failure; NYHA, New York Heart Association; TG, Training group; CG, control group; NR, Not reported; HIIT, high-intensity interval training; CRT, circuit-resistance training; HR, heart rate; VO_2max, maximum oxygen uptake; ET, exercise training; MCT, moderate continuous training; AIT, aerobic interval training; MICT, moderate intensity continuous training; HIT, high-Intensity Training; CT, continuous Training.

Table S3. Results of meta-regression.

Modalities	_ES	Coef.	Std. Err	t	p > |t|	95% CI
Intervention duration	subgroup	-0.1118706	0.0707989	-1.58	0.133	-0.2612433, 0.037502
	_cons	2.48447	0.8815045	2.82	0.012	0.6246579, 4.344282
Frequency	subgroup	0.6784313	0.2203159	3.08	0.007	0.2136055, 1.143257
	_cons	-1.053732	0.7431752	-1.42	0.174	-2.621694, 0.5142306
Session duration	subgroup	0.0064256	0.023188	0.28	0.785	-0.0429986, 0.0558497
	_cons	0.9414515	1.11998	0.84	0.414	-1.445729, 3.328632
Weekly time	subgroup	0.0126747	0.0054695	2.32	0.035	0.0010167, 0.0243327
	_cons	-0.7316918	0.883191	-0.83	0.420	-2.614169, 1.150785

Abbreviations: Coef, coefficient; Std. Err, standard error; t, t-test statistic; p, probability; CI, confidence interval.

Table S4. Results of Egger's test.

Std_EFF	Coef.	Std. Err.	t	p > |t|	95% CI
slope	-0.6926431	0.7102434	-0.98	0.343	-2.191126, 0.8058395
bias	3.531321	1.504033	2.35	0.031	0.3580879, 6.704554

Abbreviations: FMD, flow-mediated dilation; Coef, coefficient; Std. Err, standard error; t, t-test statistic; p, probability; CI, confidence interval.

Table S5. GRADE summary of evidence.

Certainty assessment							No. of participants		Effect		Certainty	Importance
No. of studies	Study design	Risk of bias	Inconsistency	Indirectness	Imprecision	Other considerations	Experimental	Control	Relative (95% CI)	Absolute		
11	RCT	low risk	no serious inconsistency	no serious indirectness	no serious imprecision	none	224	185	-	SMD 1.14 higher (0.63 to 1.66 higher)	⊕⊕⊕⊕ High	-

Note: RCT: randomized controlled trial, SMD: standardized mean difference, CI: confidence interval.
GRADE Working Group grades of evidence: ⊕⊕⊕⊕, High certainty: we are very confident that the true effect lies close to that of the estimate of the effect; ⊕⊕⊕○, Moderate certainty: we are moderately confident in the effect estimate: the true effect is likely to be close to the estimate of the effect, but there is a possibility that it is substantially different; ⊕⊕○○, Low certainty: our confidence in the effect estimate is limited: the true effect may be substantially different from the estimate of the effect; ⊕○○○, Very low certainty: we have very little confidence in the effect estimate: the true effect is likely to be substantially different from the estimate of effect.
